# The effects of two combined methods of P53 expression and preoperative serum CEA detection on the prognosis of colorectal cancer

**DOI:** 10.3389/fonc.2025.1590836

**Published:** 2025-07-21

**Authors:** Guojun Tong, Yanyan Wang, Hai Qian, Zhenhua Tan, Yan Shen, Hui Li

**Affiliations:** ^1^ Colorectal Surgery of Huzhou Central Hospital, Affiliated Central Hospital of Huzhou University, Huzhou, China; ^2^ Central Laboratory of Huzhou Central Hospital, Affiliated Central Hospital of Huzhou University, Huzhou, China; ^3^ Pathologic Department of Huzhou Central Hospital, Affiliated Central Hospital of Huzhou University, Huzhou, China; ^4^ General Surgery of Huzhou Central Hospital, Affiliated Central Hospital of Huzhou University, Huzhou, China

**Keywords:** p53, CEA, CRC, OS, DFS, prognosis, ROC

## Abstract

**Aim:**

To explore the effects of two combined methods—P53 expression and preoperative serum carcinoembryonic antigen (S-CEA) detection—on the prognosis of colorectal cancer (CRC).

**Methods:**

Two classified combinations of tissue P53 and S-CEA were utilized: Combined P53 groups (normal P53 and S-CEA, or one or both elevated) and Recombined groups (P53 normal & S-CEA normal, P53 normal & S-CEA high, P53 high & S-CEA normal, P53 high & S-CEA high). Clinicopathologic features were analyzed by P53, S-CEA, Combined P53, and Recombined P53. Correlations between them were examined. Overall survival (OS) and disease-free survival (DFS) were evaluated using the Kaplan-Meier method and Log-Rank test. Univariate and multivariate analyses were performed for Combined P53 and Recombined P53 to determine independent factors. Three-year, two-year, and one-year OS and DFS were further analyzed using multimeROC. SPSS 27 and R 4.4.1 were used for analysis.

**Results:**

TNM stage, CA199, differentiation, tumor maximum size, and minimum size showed significant differences between the single P53 and S-CEA groups (all P < 0.05). TNM stage, CA199, and chemotherapy differed in both Combined P53 and Recombined P53 groups (all P < 0.05). Significant correlations were found between P53, S-CEA, Combined P53, and Recombined P53 (all P < 0.001). No significant differences in OS and DFS were observed with P53 and Combined P53 (all P > 0.05), but differences were noted with S-CEA and Recombined P53 (all P < 0.05). Univariate and multivariate analyses identified laparoscopy, chemotherapy, differentiation, TNM stage, and Recombined P53 as independent factors for OS and DFS, while P53, S-CEA, and Combined P53 were not. Further multimeROC analysis showed that 3-year OS had better sensitivity and specificity (Area Under Curve [AUC] = 0.54), and 1-year DFS was better (AUC = 0.59).

**Conclusions:**

Recombined P53 classification was more effective than traditional Combined P53 classification for assessing CRC prognosis and was an independent factor. Additionally, the 3-year OS and 1-year DFS analysis demonstrated higher sensitivity and specificity with Recombined P53.

## 1 Introduction

Colorectal carcinoma (CRC) is the fourth leading cause of cancer-related death, accounting for 600,000 deaths annually (1). Clinical factors such as tumor stage, tumor necrosis, vascular invasion, differentiation, Ki67, serum CEA, and inflammation have been reported to influence the prognosis of CRC patients (2–5). Although significant progress has been made in understanding CRC pathogenesis and clinical treatment, tumor resection remains the preferred option, with a 5-year survival rate of less than 65% (6). The p53 gene is a critical tumor suppressor activated by DNA damage, oxidative stress, and oncogene activation to produce p53 protein, which induces DNA repair, apoptosis, and regulates cell cycle checkpoints. TP53 mutations result in the loss of tumor suppressor function, enhancing tumor invasiveness and metastasis, leading to reduced survival rates (7–10). Patients with metastatic right-sided CRC (RCC) tend to have poorer survival compared to those with left-sided CRC (LCC), particularly among those with non-gain-of-function (non-GOF) mutp53. Conversely, gain-of-function (GOF) mutp53 is associated with worse survival only in patients with LCC (11). However, a recent study found no correlation between the TP53 Arg72Pro polymorphism and CRC risk, with no significant differences in genotype and allele frequencies across sex, age, histological grade, tumor stage, smoking status, or alcohol consumption (12). Basic studies have shown that p53 suppresses tumor progression through the p53 gene and signaling pathway (13, 14). Clinical studies on p53 expression in CRC are limited and often controversial, with available evidence failing to support p53 as a prognostic marker in metastatic CRC. Prospective studies with larger sample sizes and standardized methodologies are needed to explore the prognostic role of p53 in metastatic CRC patients (15–17). Consequently, combining p53 with other tumor markers in CRC, such as S-CEA, Ki67, MLH1, p16INK4a, and Kras, has been suggested (18–22). However, the outcomes remain controversial. Huang et al. found that patients with high preoperative serum CEA levels and P53 gene mutations had poor prognoses (23). Due to CRC’s highly heterogeneous nature, a single tumor marker is unlikely to serve as a standalone diagnostic tool due to its insufficient sensitivity and/or specificity. A combined approach using multiple tumor markers for CRC diagnosis holds potential as an effective strategy. The use of serum protein biomarkers may lead to the development of inexpensive, noninvasive tests for CRC detection, and combining tumor markers could improve screening effectiveness. If serum p53 antibody levels remain elevated long after resection, the case warrants intensive follow-up (24, 25). Given the importance of joint detection of P53 in CRC patients and the ongoing controversy surrounding related research, this study aims to explore the prognostic roles of CRC through two combined methods—tissue p53 and S-CEA.

## 2 Materials and methods

### 2.1 Patients

Data were collected from 750 CRC surgery patients at our hospital between January 2017 and December 2019. A total of 265 cases were excluded due to missing clinical, pathological, or follow-up data, endoscopic resection (EMR), or death from non-tumor-related causes. Ultimately, 485 patients were included in this study. The inclusion criteria were as follows: patients diagnosed with CRC through colonoscopy, computed tomography (CT), and pathological tests, either in our hospital or elsewhere; no preoperative adjuvant treatment; surgery performed in our department; routine preoperative S-CEA detection; lymph node dissection with ≥12 lymph nodes detected (although a small number of samples with 8–11 lymph nodes were included); CRC-related death as the termination event; and postoperative routine immunohistochemical (IHC) analysis and pathological examination for P53, with postoperative chemotherapy determined according to AJCC-8 guidelines (2). The exclusion criteria included serious diseases of the heart, brain, liver, or lungs that contraindicated surgery, non-CRC tumors leading to patient death, and missing follow-up or clinicopathological data. Data bias was minimized as much as possible through these inclusion and exclusion criteria.

### 2.2 Follow up

Patients were followed up every 3 months during the first year after primary CRC surgery, then every 6 months during the second year, and annually for the remaining 3 years, for a total of 5 years. All follow-up data were obtained from our records, either by phone or through the inpatient electronic medical record system (Haitai Software Version 3.0, Nanjing). Survival time was calculated from the date of primary surgery to the date of death or the end of the follow-up period, which lasted at least 5 years. If survival exceeded 60 months, it was capped at 60 months. Death due to the primary tumor or tumor-related disease was considered a positive event, while other causes were treated as censoring events. Thus, OS and DFS were analyzed.

### 2.3 Detection of S-CEA

Venous blood was drawn from each patient before surgery, and a fully automated chemiluminescence microsphere immunoassay device (ARCHITECT) was used to detect S-CEA. A reference range of 0–5 ng/ml was considered normal, while levels >5 ng/ml were classified as high.

### 2.4 Detection of tumor P53 by immunohistochemistry

**Figure 1:**
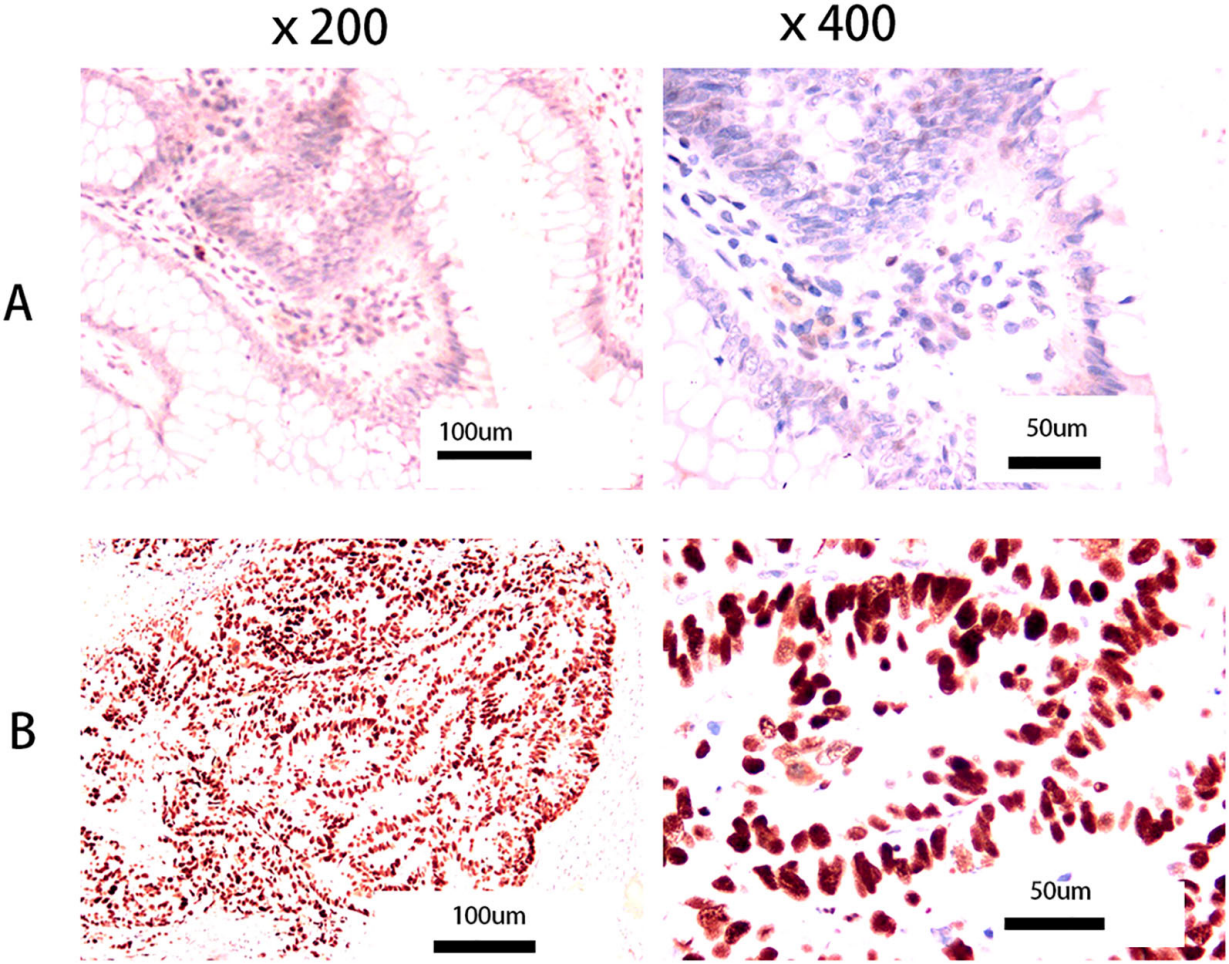
Pictures of p53 tumor expression. **(A)** Normal p53 expression (left: × 200, right: ×400); **(B)** High p53 expression (left: ×200, right: ×400). No positive cells or positive cells <5% are negative, positive cells 5%– 25% are weakly positive (+), 25% ~75% is positive (++), >75% is strong positive (+++). We refer to patients with p53 staining greater than 75% as the p53 high- expression group, and the rest as the normal group (6).

Slicing - dewaxing to water - antigen repair (citric acid thermal repair) -4% hydrogen peroxide - addition of 1 antibody (P53) - incubation at 37°C for 30 minutes - universal type 2 antibody - incubation at 37°C for 30 minutes - DAB staining for 5 minutes - dehydration and sealing. Kits were purposed from Fuzhou Maixin Biotechnology Development Co., Ltd (Kits Number: MAB0674). The criterion for p53 positive staining results is the presence of yellow or brown or tan particles in the nucleus. No positive cells or positive cells <5% are negative, positive cells 5%– 25% are weakly positive (+), 25% ~75% is positive (++), >75% is strong positive (+++). Patients with p53 staining greater than 75% were classified as the p53 high-expression group, while the remaining patients were classified as the normal group (6). **Figure 1A** shows normal p53 expression (left ×200, right ×400), and **Figure 1B** shows high p53 expression (left ×200, right ×400).

### 2.5 Two methods of combined P53 and S-CEA Classification (Combined P53 and Recombined P53)

Based on previous literature (26), when both p53 and S-CEA are normal, the classification is considered normal; otherwise, it is considered high, resulting in two groups: Combined P53: normal and high. Another method, as defined in two previously published studies (27, 28) (including our own), is the Recombined P53 method, which divides patients into four groups: p53 normal & S-CEA normal, p53 normal & S-CEA high, p53 high & S-CEA normal, and p53 high & S-CEA high.

### 2.6 Receiver operating characteristic curve analysis

OS and DFS were analyzed using ROC curves for Recombined P53. ROC curves were carried out for 1, 2, and 3 years of OS and DFS, and the sensitivity and specificity for these time periods were analyzed based on the AUC values.

### 2.7 Statistical analysis

All clinicopathological features were analyzed using SPSS 27. ANOVA and crosstab methods were employed to analyze continuous and categorical variables, respectively. Means and standard deviations were calculated. Comparisons of clinicopathological features between p53, S-CEA, combined p53, and recombined p53 were performed using the Tukey and χ² tests. Kaplan–Meier and log-rank tests were used for survival analysis between groups. Cox regression analysis was performed for univariate and multivariate analyses. MultiTimeRoc and five-year OS and DFS survival curves with numbers at risk were generated using R software (version 4.4.1) with the “ggplot2,” “survival,” “survminer,” and “timeROC” packages.

## 3 Results

### 3.1 Clinicopathological features in p53, S-CEA, combined p53, recombined p53

A total of 485 cases were included, with a mean age of 65.13 years and a standard deviation (SD) of 10.84 (ranging from 25 to 90). Significant differences in gender, TNM stage, Carbohydrate Antigen 199 (CA199), differentiation, and both minimum and maximum tumor size were observed in the single p53 groups (all P<0.05). However, no significant differences were found for age, laparoscopy, tumor location, duration (days), lymph node harvest, costs, preoperative C-reactive protein (CRP), preoperative albumin, postoperative chemotherapy, or complications (all P>0.05) in the single p53 groups. Significant differences in TNM stage, CA199, postoperative chemotherapy, differentiation, and both minimum and maximum tumor size were observed in the single S-CEA groups (all P<0.05), while other variables showed no significant differences (all P>0.05). Significant differences in TNM stage, CA199, and postoperative chemotherapy were found in the combined p53 groups (all P<0.05), while other variables showed no significant differences (all P>0.05). In the recombined p53 groups, significant differences were noted for tumor location, TNM stage, CA199, postoperative chemotherapy, and both minimum and maximum tumor size (all P<0.05), while other variables showed no significant differences (all P>0.05). The detailed values and comparisons are shown in **Table 1**.

**Table 1:** Clinicopathologic features compared by P53, S-CEA, combined P53, recombined P53(N,%,Mean,SD).

Variations	P53 expression		P	S-CEA expression		P	Combined P53		P	Recombined P53				P
	Normal(n=263)	High(n=222)		Normal(n=283)	High(n=202)		Normal(n=162)	High(n=323)		P53normal&S-CEA normal (n=162)	P53normal&S-CEA high(n=101)	P53high&S-CEAnormal (n=121)	P53high&S-CEA high(n=101)	
Gender			0.04*			0.83								0.22
M	144(29.7)	142(29.3)		168 (34.4)	118 (24.3)		89(18.4)	197(40.6)	0.20	89(18.4)	55(11.3)	79(16.3)	63(13.0)	
F	119(24.5)	80(16.5)		115(23.7)	84(17.3)		73(15.1)	126(26.0)		73(15.1)	46(9.5)	38(7.8)	43(8.9)	
Age(year)	65.12(11.4)	65.14(10.1)	0.98	64.78(10.7)	65.62(11.0)	0.40	65.35(11.2)	65.02(10.7)	0.76	65.35(11.2)	64.76(11.9)	64.03(10.1)	66.48(10.1)	0.40
Laparoscopy			0.30			0.06			0.82					0.15
Y	155(32.0)	141(29.1)		183(37.7)	113(23.3)		100(20.6)	196(40.4)		100(20.6)	55(11.3)	83(17.1)	58(12.0)	
N	108(22.3)	81(16.7)		100(20.6)	89(18.4)		62(12.8)	127(26.0)		62(12.8)	46(9.5)	38(7.8)	43(8.9)	
Location			0.06			0.31			0.08a					<0.001a***
ileocecus	20(4.1)	10(2.1)		19(3.9)	11(2.3)		13(2.7)	17(3.5)		13(2.7)	7(1.4)	6(1.2)	4(0.8)	
right colon	37(7.6)	20(4.1)		28(5.8)	29(6.0)		21(4.3)	36(7.4)		21(4.3)	16(3.3)	7(1.4)	13(2.7)	
transverse colon	30(6.2)	16(3.3)		33(6.8)	13(2.7)		21(4.3)	25(5.2)		21(4.3)	9(1.9)	12(2.5)	4(0.8)	
sigmoid colon	46(9.5)	49(10.1)		55(11.3)	40(8.2)		28(5.8)	67(13.8)		28(5.8)	18(3.7)	27(5.6)	22(4.5)	
left colon	7(1.4)	12(2.5)		10(2.1)	9(1.9)		2(0.4)	17(3.5)		2(0.4)	5(1.0)	8(1.6)	4(0.8)	
rectum	123(25.4)	115(23.7)		138(28.5)	100(20.6)		77(15.9)	161(33.2)		77(15.9)	46(9.5)	61(12.6)	54(11.1)	
Duration(days)	22.44(11.1)	22.36(8.2)	0.93	22.12(8.4)	22.80(11.7)	0.46	21.86(7.8)	22.67(10.8)	0.40	21.86(7.8)	23.37(15.0)	22.47(9.1)	22.23(7.2)	0.69
Lymph harvest(n)	15.43(6.1)	14.76(10.7)	0.39	14.62(6.1)	15.82(11.1)	0.65	15.28(5.6)	16.04(9.7)	0.35	15.28(5.6)	15.65(6.9)	13.73(6.7)	15.99(14.1)	0.19
Cost(CNY, 10thouthend)	4.21(0.97)	4.33(1.2)	0.21	4.28 (1.1)	4.24 (1.1)	0.65	4.26(1.0)	4.26(1.1)	0.98	4.26(1.0)	4.11(0.96)	4.30(1.3)	4.36(1.1)	0.40
TNM			<0.001*			<0.001***			0.002**					<0.001a***
0&I	53(10.9)	45(9.3)		75(15.5)	23(4.7)		41(8.5)	57(11.8)		41(8.5)	12(2.5)	34(7.0)	11(2.3)	
II	104(21.4)	64(21.6)		101(20.8)	67(13.8)		67(13.8)	101(20.8)		67(13.8)	37(7.6)	34(7.0)	30(6.2)	
III	91	10.5(20.6)		97(20.0)	99(20.4)		46(9.5)	150(30.9)		46(9.5)	45(9.3)	51(10.5)	54(11.1)	
IV	15(3.1)	8(1.6)		10(2.1)	13(2.7)		8(1.6)	15(3.1)		8(1.6)	7(1.4)	2(0.4)	6(1.2)	
CRP(mg/L)	7.66 (16.9)	7.43(21.1)	0.89	7.94 (20.9)	7.01 (15.6)	0.60	7.50(16.2)	7.58(20.1)	0.97	7.50(16.2)	7.90(18.0)	8.51(26.1)	6.13(12.7)	0.82
Albumin(g/L)	37.71(4.8)	37.9(5.2)	0.63	37.79 (4.8)	37.83 (5.2)	0.93	37.59(4.9)	37.91(5.0)	0.49	37.59(4.9)	37.90(4.6)	38.06(4.7)	37.76(5.8)	0.88
CA199(U/ml)	94.66(464.1)	33.06(104.6)	0.04*	12.43 (34.0)	142.16(532.4)	<0.001***	12.12(24.9)	93.72(426.1)	<0.001***	12.12(24.9)	227.06(731.1)	12.86(43.4)	57.26(144.3)	<0.001***
Postoperative chemotherapy			0.17			<0.001***			0.032*					0.008**
N	107(22.1)	82(16.9)		128(26.4)	61(12.6)		74(15.3)	115(23.7)		74(15.3)	33(6.8)	54(11.1)	28(5.8)	
Y	156(32.2)	140(28.9)		155(32.2)	141(29.1)		88(18.1)	208(42.9)		88(18.1)	68(14.0)	67(13.8)	73(15.1)	
Differentiation			<0.001***			0.005**			0.20					
poor & undifferentiation	121(24.9)	71(14.6)		95(19.6)	97(20.0)		62(12.8)	130(26.8)		62(12.8)	59(12.2)	33(6.8)	38(7.8)	
moderate	133(27.4)	130(26.8)		167(34.4)	96(19.8)		94(19.4)	169(34.8)		94(19.4)	39(8.0)	73(15.1)	57(11.8)	
high	9(1.9)	21(4.3)		21(4.3)	9(1.9)		6(1.2)	24(4.9)		6(1.2)	3(0.6)	15(3.1)	6(1.2)	
Complication			0.58			0.433			0.82					0.21
N	243(50.1)	202(41.6)		262(54.0)	183(37.7)		148(30.5)	297(61.2)		148(30.5)	95(19.6)	114(23.5)	88(18.1)	
Y	20(4.1)	20(4.1)		21(4.3)	19(3.9)		14(2.9)	26(5.4)		14(2.9)	6(1.2)	7(1.4)	13(2.7)	
Tumor size														
Maximum(cm)	4.71(1.9)	4.09(2.0)	<0.001***	4.19 (1.9)	4.75 (1.9)	0.002**	4.60(1.9)	4.33(1.9)	0.16	4.60(1.9)	4.87(1.78)	3.65(1.8)	4.62(2.0)	<0.001***
Minimum(cm)	3.32(1.5)	2.87(1.6)	0.002**	2.93(1.5)	3.37(1.5)	0.002**	3.18(1.4)	3.08(1.6)	0.50	3.18(1.4)	3.54(1.6)	2.60(1.6)	3.20(1.5)	<0.001***

*P<0.05;**P<0.01;***P<0.001;NA:not available.a:Fisher’s exact test.

### 3.2 Comparisons of 5 year overall survival rate, disease-free survival rate by p53,S-CEA,combined p53, recombined p53 groups

**Figure 2:**
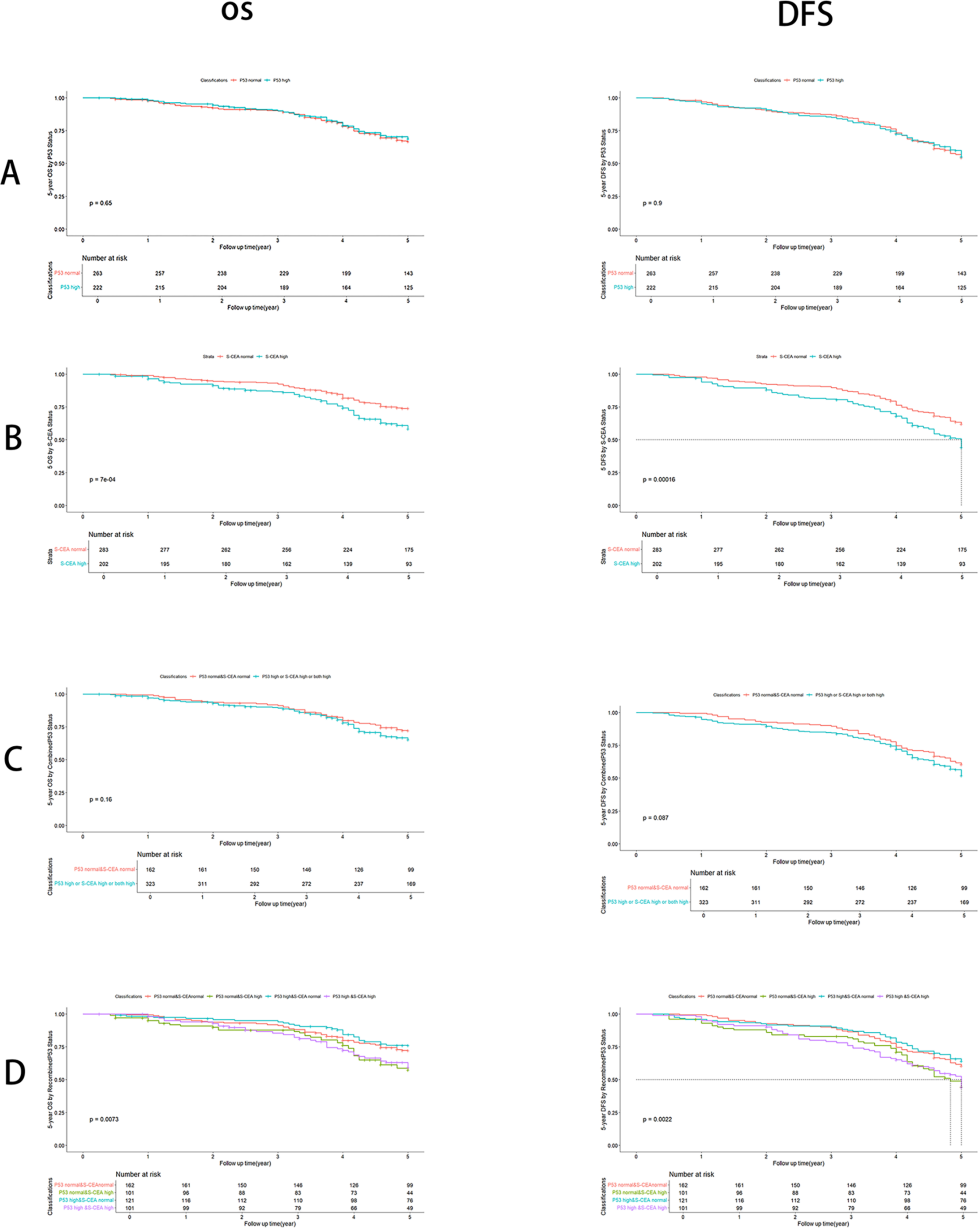
Overall survival (OS) and Disease free survival (DFS) analysis with number at risk by Kaplan-Meier and Log-rank test using R 4.4.1. **(A)** By p53 groups, there are no significant differences about OS (P=0.65) and DFS (P=0.9); **(B)** By S-CEA groups, there are significant differences about OS (P<0.001) and DFS (P<0.001); **(C)** By combined p53 groups, there are no significant differences about OS (P=0.16) and DFS (P=0.087); **(D)** By recombined p53, there are significant differences about OS (P=0.0073) and DFS (P=0.0022).

There were no significant differences in OS and DFS rates between the single p53 groups (P=0.65 and P=0.90, respectively, **Figure 2A**). Significant differences in OS and DFS rates were observed in the single S-CEA groups (both P<0.001, **Figure 2B**). No significant differences in OS and DFS rates were found in the combined p53 groups (P=0.16 and P=0.087, respectively, **Figure 2C**). However, significant differences in OS and DFS rates were observed in the recombined p53 groups (P=0.0073 and P=0.0022, **Figure 2D**).

### 3.3 Correlations between P53, S-CEA, combined P53 and recombined P53

The Pearson correlation between p53 and S-CEA was 0.072 (P=0.057, one-tailed), with no significant difference. The Pearson correlations between p53 and combined p53, as well as p53 and recombined p53, were 0.651 and 0.903, respectively, both showing significant differences (both P<0.001, one-tailed). The Pearson correlations between S-CEA and combined p53, as well as S-CEA and recombined p53, were 0.598 and 0.494, respectively, both significant (P<0.001, one-tailed). The Pearson correlation between combined p53 and recombined p53 was 0.825 (P<0.001, one-tailed). The details are shown in **Table 2**.

**Table 2:** Correlations between P53,S-CEA, combined P53 and recombined P53.

	P53	S-CEA	Combined P53	Recombined P53
P53				
Pearson correlation	1	0.072	0.651	0.903
Significance(one- tailed)		0.057	<0.001***	<0.001***
N	485			
S-CEA				
Pearson correlation	0.072	1	0.598	0.494
Significance(one- tailed)	0.057		<0.001***	<0.001***
N	485	/		
Combined P53				
Pearson correlation	0.651	0.598	1	0.825
Significance(one-tailed)	<0.001***	<0.001***		<0.001***
N	485			
Recombined P53				
Pearson correlation	0.903	0.494	0.825	1
Significance(one-tailed)	<0.001***	<0.001***	<0.001***	
N	485			

*P<0.05;**P<0.01; ***P<0.001.

### 3.4 Univariate and multivariate analysis of overall survival and disease free survival

The categorical variables were analyzed by univariate and multivariate analyses for OS and DFS using Cox regression. Variables that showed significant differences in univariate analysis were further analyzed by multivariate analysis. The method used was the input approach, with the first factor as the reference. For OS, significant differences were found in location, laparoscopy, postoperative chemotherapy, differentiation, TNM stage, S-CEA, and recombined p53 (all P<0.05). However, no significant differences were observed for gender, p53, or combined p53 in the univariate analysis. In the multivariate analysis, only laparoscopy, postoperative chemotherapy, differentiation, TNM stage, and recombined p53 showed significant differences, indicating that they are independent factors for OS. For DFS, significant differences were found in laparoscopy, postoperative chemotherapy, differentiation, TNM stage, S-CEA, and recombined p53 in the univariate analysis. In the multivariate analysis, only laparoscopy, postoperative chemotherapy, differentiation, TNM stage, and recombined p53 remained significant, indicating that they are independent prognostic factors for CRC. Based on these analyses, recombined p53 emerged as an independent factor for CRC prognosis in both OS and DFS, while single p53, S-CEA, and combined p53 were not. **Table 3** shows the details.

**Table 3:** Univariate and Multivariate analysis of overall survival (OS)and disease free survival(DFS).

Variables	OS				DFS			
	Univariate	P value	Multivariat	P value	Univariate	P value	Multivariat	P value
	HR(95%CI)		HR(95%CI)		HR(95%CI)		HR(95%CI)	
Gender		0.662				0.121		
m	1				1			
f	1.076(0.774-1.495)				1.236(0.946-1.617)			
Location		0.015*		0.427		0.078		
ileocecus	1		1		1			
right colon	0.748(0.383-1.462)		0.639(0.314-1.264)		0.831(0.450-1.536)			
transverse colon	0.801(0.401-1.597)		1.026(0.506-2.080)		0.880(0.468-1.657)			
sigmoid colon	0.629(0.333-1.186)		0.910 (0.474-1.745)		0.889(0.505-1.562)			
left colon	0.500(0.180-1.390)		0.507(0.177-1.451)		0.694(0.297-1.621)			
rectum	0.414(0.230-0.744)		0.703(0.373-1.324)		0.576(0.338-0.980)			
Laparoscopy		<0.001***		0.014*		<0.001***		0.002**
yes	1		1		1		1	
no	1.914(1.383-2.649)		1.573(1.095-2.259)		1.790(1.370-2.338)		1.569(1.185-2.078)	
Chemotherapy		<0.001***		<0.001***		<0.001***		0.003*
no	1		1		1		1	
yes	1.917(1.332-2.759)		0.445(0.291-0.680)		1.958(1.451-2.642)		0.586(0.412-0.834)	
Differentiation		<0.001***		<0.001***		<0.001***		<0.001***
poor & un	1		1		1		1	
moderate	0.313(0.222-0.440)		0.405(0.281-0.584)		0.395(0.301-0.520)		0.511(0.385-0.678)	
high	0.099(0.024-0.402)		0.314(0.072-1.360)		0.106(0.034-0.333)		0.242(0.073-0.797)	
TNM		<0.001***		<0.001***		<0.001***		<0.001***
0&I	1		1		1		1	
II	6.144(2.190-17.239)		6.912(2.326-20.539)		3.192(1.713-5.949)		2.987(1.526-5.848)	
III	15.219(5.578-41.527)		20.716(6.876-62.417)		7.863(4.344-14.233)		8.218(4.108-16.440)	
IV	82.007(27.905-241.001)		100.568(30.799-328.324)		32.006(15.644-65.481)		31.154(13.767-70.496)	
P53 expression		0.653				0.897		
normal	1				1			
high	0.928(0.669-1.286)				0.983			
S-CEA		<0.001***		0.327		<0.001***		0.421
normal	1		1		1		1	
high	1.737(1.255-2.403)		1.263(0.792-2.013)		1.655(1.267-2.161)		1.164(0.804-1.687)	
Combined P53		0.166				0.091		
normal	1				1			
high	1.286(0.901-1.836)				1.287(0.961-1.732)			
Recombined P53		0.009**		0.031*		0.003**		0.026**
P53normal &CEAnormal	1		1		1		1	
P53normal &CEAhigh	1.637(1.061-2.525)		0.856(0.534-1.374)		1.557(1.084-2.238)		0.924(0.629-1.358)	
P53high &CEAnormal	0.825(0.510-1.335)		0.905(0.547-1.374)		0.883(0.600-1.299)		0.875(0.591-1.297)	
P53high &CEAhigh	1.574(1.014-2.443)		1.2.8(0.789-2.445)		1.585(1.105-2.272)		1.279(1.038-2.216)	

*P<0.05;**P<0.01;**P<0.001; Cox regression was used for univariate and multivariate analysis. Variables which have significant difference by univariate analysis were analyzed by multivariate analysis.

### 3.5 1,2,3 years of receiver operating characteristic curve analysis by recombined p53

**Figure 3:**
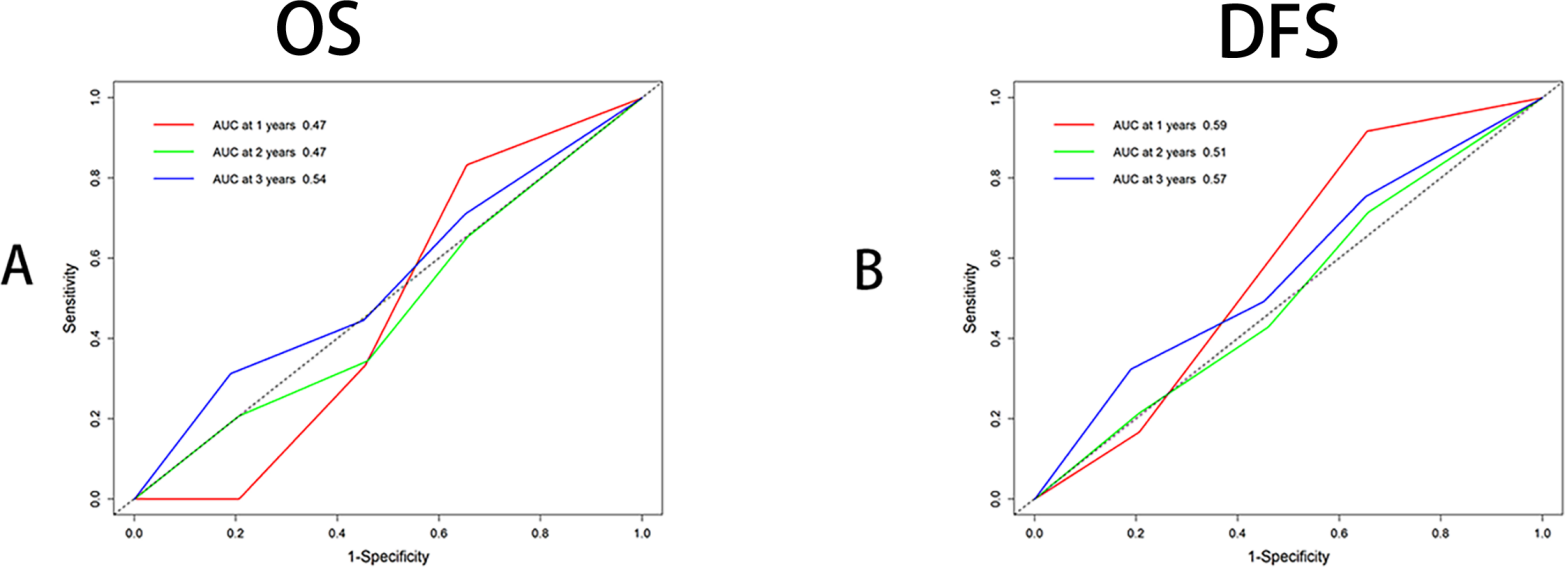
Receiver operating characteristic curve analysis (ROC) by multitimeROC using R4.4.1. **(A)** ROC analysis using OS (Binary variable), Area Under Curve (AUC) were 0.47,0.47 and 0.54 respectively for 1,2,3 years indicating 3 years has superior. **(B)** ROC analysis using DFS (Binary variable), Area Under Curve (AUC) were 0.59, 0.57 and 0.51 respectively for 1,2,3 years indicating 1 year has superior.

After identifying recombined p53 as an independent prognostic factor, we performed ROC analysis for OS and DFS at 1, 2, and 3 years. The results showed that recombined p53 demonstrated superiority in predicting 3-year OS (AUC=0.54, **Figure 3A**) and 1-year DFS (AUC=0.59, **Figure 3B**). We know that the closer the AUC value is to 1, the higher the sensitivity and specificity (29, 30).

## 4 Discussion

As we know, p53 is a tumor suppressor gene for CRC and other malignant tumors. Its mutation and deficiency often promote tumor invasion, progression, and metastasis (31–36). Many genes, such as ubiquitin-specific protease 36 (USP36) and carnitine palmitoyltransferase-2 (CPT2), affect the progression of CRC through the p53 signaling pathway (37–43). However, clinical studies on p53 have shown varying prognostic efficacy for CRC (31, 44). This study demonstrated that single p53 detection has no effect on the prognosis of CRC. Therefore, combining p53 with other genes has been a hot topic recently, such as combining p53 with mitochondrial translation elongation factor Tu (TUFM) and metastasis suppressor 23-H1 (Nm23-H1) (20, 25, 45). Preoperative and postoperative S-CEA detection are commonly used for CRC screening and prognostic recurrence assessment as reference indicators, but many CRC patients do not show elevated preoperative S-CEA levels (46–48). This study found that the preoperative S-CEA elevation rate is 41.6% (202/485), indicating its limited sensitivity. While this study showed that single S-CEA has a prognostic role in CRC, it is not an independent factor for OS and DFS, which is consistent with some published literature (49–52). Due to the controversy surrounding the limitations and prognostic impact of detecting tumor p53 and preoperative S-CEA separately for CRC, we aimed to explore the effect of combined detection on CRC prognosis using two different combinations from previous studies (26, 28, 46, 53, 54). Despite the findings regarding p53 vs S-CEA, significant pairwise associations were found among p53, S-CEA, combined p53, and recombined p53. This study showed that gender, TNM stage, Carbohydrate Antigen 199 (CA199), differentiation, and tumor size (both minimum and maximum) had significant differences in the single p53 group. TNM, CA199, postoperative chemotherapy, differentiation, and tumor size (both minimum and maximum) had significant differences in the single S-CEA group. In the combined p53 group, TNM, CA199, and postoperative chemotherapy showed significant differences, while in the recombined p53 group, tumor location, TNM, CA199, postoperative chemotherapy, and tumor size (both minimum and maximum) showed significant differences. Kaplan-Meier and Log-rank tests showed that S-CEA and recombined p53 had significant differences for OS and DFS of CRC, whereas single p53 and combined p53 showed limited effectiveness for prognostic values, which is consistent with some published literature (15, 31). However, some studies indicated that loss of p53 expression predicted a worse prognosis in CRC (55–57). Through Cox regression analysis, this study found that p53, S-CEA, and combined p53 were not independent factors for OS and DFS of CRC, while recombined p53 was, emphasizing the importance of recombined p53 for CRC prognosis. These findings underscore the limitations of single p53, S-CEA detection, and the combined p53 method, aligning with results from previous literature (11, 15, 17, 58). After identifying recombined p53 as an independent prognostic factor, we performed ROC analysis for OS and DFS at 1, 2, and 3 years. The results showed that recombined p53 demonstrated superiority in predicting 3-year OS.Du to no longer follow-up data than 5 years, we can’t analyze 1,3,5 years ROC about OS and DFS which we may mention in the limitations meanwhile the AUC value in this article is around 0.5, which may lead to insufficient persuasiveness in the AUC analysis section while one similar study showed higher AUC value (59). TNM staging remains a key factor in the prognosis of CRC, and this study further evaluated the TNM staging in AJCC-8. We did not overlook the role of TNM staging. On the contrary, this study found significant differences in TNM among CEA, P53, Combined P53, and Recombined P53 (**Tables 1**, **3**).

Due to the fact that only recombined p53 is an independent factor for OS and DFS in CRC, we performed 1-, 2-, and 3-year time ROC analysis. The results showed that recombined p53 demonstrated superior performance at 3 years for OS and at 1 year for DFS.

## 5 Limitations

This study has several limitations. These include the absence of genetic analysis, the reliance on outdated data, and the fact that it is a single-center, retrospective study. It is also important to note that Stage IV refers only to patients with resectable Stage IV colorectal carcinoma, excluding all Stage IV patients, which limits the generalizability of the findings. Additionally, the study is based on data from a single medical center, which could limit the applicability of the findings to a broader population. According to statistical principles, the follow-up period is 5 years instead of 10 years, so we can only perform ROC analysis on OS and DFS for 1, 2, and 3 years. If the follow-up period is 10 years, we can perform ROC analysis on OS and DFS for 1, 3, and 5 years. This is a limitation of our study. Genetic typing or molecular analysis of p53 is more precise than IHC staining. This study is a retrospective study in which the data came from pathological department using ICH method to type the analysis of p53 that is more general and more affordable. The AUC value in this article is around 0.5, which may lead to insufficient persuasiveness in the AUC analysis section. However, the data collected in this study shows these results. These may be another limitations in this paper.

## 6 Conclusions

Recombined p53 classification proved to be superior to traditional combined p53 classification for assessing the prognosis of CRC and was found to be an independent factor. Furthermore, the 3-year OS and 1-year DFS analysis demonstrated greater sensitivity and specificity for recombined p53.

## Data Availability

The original contributions presented in the study are included in the article/Supplementary Material. Further inquiries can be directed to the corresponding author.
